# Transcutaneous Auricular Vagus Nerve Stimulation Selectively Improves Executive Control and Cognitive Flexibility Following Sustained High Cognitive Load: A Randomized, Single-Blind, Sham-Controlled Study

**DOI:** 10.3390/brainsci16091001

**Published:** 2026-09-21

**Authors:** Haixu Lyu, Haiyan Liu, Yifan Yang, Wanying Xing, Tingwei Feng, Xufeng Liu

**Affiliations:** 1 Department of Military Medical Psychology, Air Force Medical University, Xi’an 710032, China; lvhiaxu5510@163.com (H.L.); lhy0727ap@163.com (H.L.);; 2 School of Teacher Education, Qiongtai Normal University, Haikou 571127, China

**Keywords:** taVNS, executive function, cognitive load, high cognitive load, attention networks, cognitive flexibility, neuromodulation

## Abstract

**Highlights:**

**What are the main findings?**

- A single 30 min taVNS session selectively improved executive control (reduced conflict effect) and cognitive flexibility (reduced switch cost) immediately following a 1 h high-cognitive-load dual task.
- These improvements were evident immediately post-intervention; model-estimated between-group differences remained directionally consistent over the 8 h window, but group × time interactions were nonsignificant, so the persistence of the effects cannot be established. No effects were observed on alerting, orienting, or working memory.

**What are the implications of the main findings?**

- taVNS shows potential as a portable, rapid-acting approach for improving executive function following sustained high cognitive load; current evidence is limited to healthy young men under laboratory conditions.
- The domain-selective pattern is consistent with preferential LC–NE innervation of prefrontal executive networks, although this mechanistic interpretation remains a hypothesis that was not directly tested in the present study.

**Abstract:**

Objectives: Acute exposure to sustained high cognitive load rapidly depletes executive functions, yet rapid, field-compatible countermeasures remain scarce. This study examined whether transcutaneous auricular vagus nerve stimulation (taVNS) improves cognitive functions across multiple domains—including the attention networks, cognitive flexibility, and working memory—following sustained high cognitive load. Methods: Healthy young men completed a 1 h dual task combining spatial 1-back and color–word Stroop judgments (the BS task) to induce a high-cognitive-load state; 65 participants were randomized to receive 30 min of active taVNS (left cymba conchae; 25 Hz; 500-μs pulse width; 30 s on/30 s off) or sham stimulation under a single-blind design, and 62 provided complete data and were analyzed. Cognitive performance was assessed with the Attention Network Test, Cued Switching Task, and spatial 2-back task immediately after the intervention and after 1, 2, 4, and 8 h; baseline-corrected change scores were analyzed with linear mixed-effects models. Results: The induction task reliably increased subjective workload and degraded task accuracy. Immediately after the intervention, taVNS significantly reduced the conflict effect relative to sham (*b* = −16.43 ms, 95% CI [−32.03, −0.82], *F*(1, 127.5) = 4.34, *p* = 0.039, *d* = 0.64) and the switch cost (*b* = −59.79 ms, 95% CI [−109.66, −9.92], *F*(1, 81.8) = 5.69, *p* = 0.019, *d* = 0.53); both immediate effects remained significant after Holm correction. Group × time interactions were nonsignificant, and the temporal course of the between-group differences should therefore be interpreted cautiously. No significant effects were observed for alerting, orienting, or working memory. Conclusions: These findings indicate that taVNS selectively and immediately improves executive control and cognitive flexibility following sustained high cognitive load, supporting its potential as a portable, rapid-acting cognitive countermeasure for high-demand operational settings.

## 1. Introduction

Acute exposure to sustained high cognitive load rapidly depletes executive functions, including inhibitory control and cognitive flexibility, thereby degrading task performance in high-demand operational settings [1,2]. Under conditions of extreme cognitive demand—such as military parachute jumping or sustained tactical decision-making—prolonged stress exposure overloads cognitive resources and markedly impairs inhibitory control, attention regulation, and rapid decision-making [3,4]. Specifically, sustained engagement in demanding cognitive tasks (e.g., 50 min Stroop interference) degrades conflict resolution and inhibitory control, even when performed in isolation without physical exertion [5]. Neurobiologically, prolonged cognitive exertion is associated with the accumulation of metabolic byproducts in the prefrontal cortex and anterior cingulate cortex, which compromises effort valuation and cognitive control [6]. These cognitive decrements not only diminish individual operational effectiveness but may also compromise collective mission efficacy.

Existing countermeasures for acute cognitive depletion are limited: pharmacological agents (e.g., psychostimulants) carry risks of dependency and adverse effects that limit their applicability in operational settings [7], whereas cognitive training requires extended implementation and sustained motivation, rendering it incompatible with acute recovery needs [8,9]. Non-invasive brain stimulation techniques (e.g., tDCS and rTMS) show cognitive enhancement potential, but their field deployment is limited by equipment size and the need for specialized administration [10]. Consequently, rapid-acting, portable, and self-administrable interventions that improve executive function following sustained high cognitive load are urgently needed.

Transcutaneous auricular vagus nerve stimulation (taVNS) has emerged as a promising non-invasive neuromodulation approach for cognitive enhancement. By delivering low-intensity microcurrents to the auricular branch of the vagus nerve, taVNS modulates neural network dynamics via ascending central–peripheral pathways, which has been proposed to act in part through the locus coeruleus–norepinephrine (LC–NE) system [11,12,13]. Relative to transcranial magnetic stimulation (TMS) and transcranial direct current stimulation (tDCS), taVNS offers distinct operational advantages: it is highly portable, is safe, and imposes minimal compliance demands [14,15]. Notably, prior studies have demonstrated that taVNS can enhance attention, memory consolidation, and executive function in healthy populations without prior exposure to high cognitive load [16,17,18]. Crucially, emerging evidence indicates that taVNS effects are amplified under high cognitive demand: taVNS significantly improves spatial working memory under high-load conditions (e.g., 3-back tasks), whereas no effect is observed in low-load tasks (e.g., 1-back) [19], and it selectively reduces switch costs under complex task-switching conditions [20]. These findings suggest that taVNS may be particularly effective when cognitive resources are heavily taxed. However, prior work has demonstrated taVNS-induced enhancement in the absence of high cognitive load; whether taVNS can improve cognitive function following acute exposure to high cognitive load—the scenario most germane to high-demand operations—has received little direct empirical attention.

Specifically, three questions remain unanswered. The first concerns whether taVNS can improve executive control and cognitive flexibility following sustained high cognitive load. The second concerns whether such improvements extend to other cognitive domains (e.g., alerting, orienting, and working memory). The third concerns the temporal dynamics of this effect, namely, whether it is immediate, sustained, or transient. The present study addresses these questions by employing a validated dual-task paradigm combining spatial 1-back and color–word Stroop judgments (the BS task) to induce a high-cognitive-load state, followed by randomized, single-blind, sham-controlled taVNS intervention [21]. We hypothesized that taVNS would improve performance across cognitive domains immediately post-stimulation, including executive control, cognitive flexibility, alerting, orienting, and working memory. The primary objective was to systematically examine the effects of taVNS on the attention networks (alerting, orienting, and executive control), cognitive flexibility, and working memory following acute exposure to high cognitive load, and to characterize the temporal dynamics of these effects.

## 2. Methods and Measures

### 2.1. Participants

Sixty-five healthy young male adults were recruited via convenience sampling. All participants were right-handed, with normal or corrected-to-normal vision, and reported no history of neurological disorders, psychiatric conditions, or chronic medication use. Only male participants were recruited because the target application population—personnel in high-demand operational settings—is predominantly male, as well as to avoid potential confounding of taVNS effects by menstrual-cycle-related hormonal fluctuations; the implications of this decision for generalizability are addressed in the Discussion. Written informed consent was obtained from all participants prior to enrollment. The study protocol was approved by the Ethics Committee of the First Affiliated Hospital of Air Force Medical University and conducted in accordance with the Declaration of Helsinki. Because no prior study had examined the effects of taVNS on cognition following acute high cognitive load, a defensible a priori effect size was unavailable; the sample size was therefore justified using a sensitivity approach [22]. A sensitivity power analysis was conducted using G*Power 3.1 (Heinrich-Heine-University Düsseldorf, Düsseldorf, Germany) (repeated-measures ANOVA, between-factor effect; 2 groups × 5 measurements; α = 0.05; power = 0.80; correlation among repeated measures = 0.50). The final sample (*N* = 62) provides 80% power to detect between-group effects of *f* ≥ 0.28 (corresponding to *d* ≈ 0.56). Sixty-five participants were randomized (33 to taVNS, 32 to sham). Three participants were excluded at the baseline stage—one in the taVNS group owing to a common cold during baseline testing, and two in the sham group (one owing to a common cold during baseline testing; one owing to concurrent medication use)—yielding a final valid sample of 62 participants (32 taVNS, 30 sham; mean age = 23.72 ± 2.47 years), with complete data at all five post-intervention assessments (310 observations). A participant flow diagram is provided in the Supplementary Materials (Figure S2).

### 2.2. Design and Procedure

**Figure 1:**
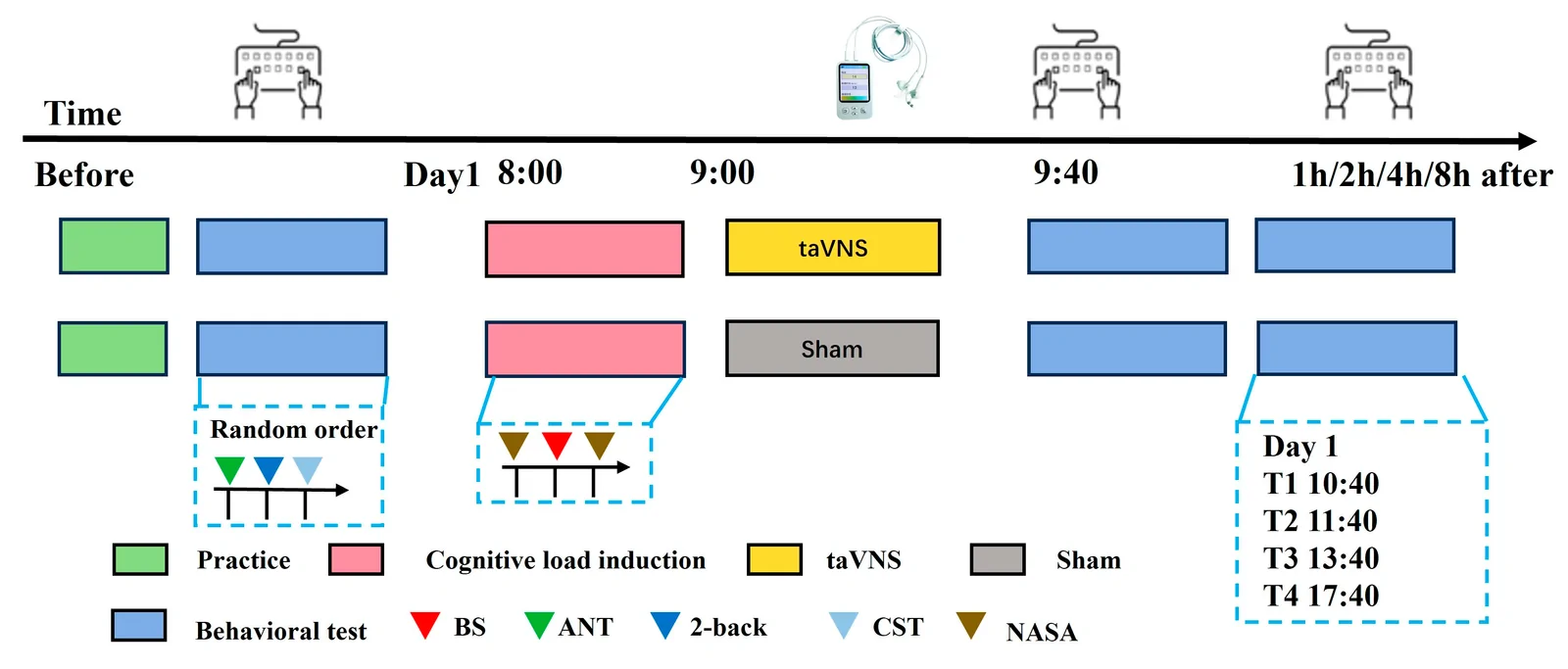
Overview of the study. BS = the 1-back Stroop high-cognitive-load task; ANT = Attention Network Test; 2-back = spatial 2-back task; CST = Cued Switching Task; NASA-TLX = NASA Task Load Index. Participants were randomly assigned to the taVNS group or the sham group (electrodes placed at the same stimulation site; current was briefly delivered to mimic the initial sensation and then turned off).

One week prior to the formal experiment, participants completed practice sessions for all behavioral tasks and baseline data collection, scheduled at a comparable time of day as the experimental session. Participants were instructed to avoid staying up late the night before each testing session and to refrain from coffee, energy drinks, and other stimulating beverages before testing. Baseline measurements included cognitive task performance (the Attention Network Test, Cued Switching Task, and 2-back task). Baseline data served only as the reference for computing change scores and were not entered into the main statistical analyses. On the day of the experiment, participants arrived at the laboratory at 8:00 a.m., completed the NASA-TLX (pre-task), and then performed the BS task continuously for 60 min to induce acute high cognitive load. The task was divided into six 10 min blocks with 20 s breaks between blocks, during which participants were instructed to remain cognitively engaged and avoid complete relaxation. Immediately after the BS task, participants completed the NASA-TLX again (post-task) to assess the induced change in subjective workload. Participants were randomly assigned to the taVNS group or the sham group at enrollment using a computer-generated random sequence with block randomization (block size = 6); enrollment was performed by two researchers, and the allocation list was held by one researcher (W.X.). Within 5 min of completing the high-cognitive-load task, the pre-allocated intervention was administered by a third-party researcher following a standardized protocol. Group allocation was concealed from participants throughout the entire study (single-blind); the researchers conducting testing and data analysis were aware of group allocation, which is acknowledged as a limitation (see Section 4). The taVNS group received transcutaneous auricular vagus nerve stimulation applied to the left cymba conchae using a portable device (tVNS501, Changzhou Rishena Medical Devices Co., Ltd., Changzhou, China; pulse width: 500 μs; frequency: 25 Hz; current intensity individually titrated to a strong but not painful; the device does not display or record absolute current values, precluding dosimetric reporting in mA), delivered in 30 s on/30 s off cycles for a total duration of 30 min. In the sham group, electrodes were placed at the identical site (left cymba conchae) and connected to the same device. Current was initially delivered and gradually increased until the participant reported a slight tingling sensation, after which the current was turned off; the electrodes and device remained in place for the full 30 min session, with identical indicator lights and device sounds throughout. This procedure reproduces the initial cutaneous sensation of active stimulation while delivering no effective stimulation, thereby maintaining participant blinding; all other procedures were identical to those of the taVNS group. At the end of the experimental session (after the final 8 h assessment), participants completed a brief stimulation-perception questionnaire (seven items rated on 0–10 scales: visual perceptual changes, acute mood changes, fatigue relief, headache, difficulty concentrating, perceived stimulation intensity, and the expected influence of the stimulation on subsequent performance); between-group comparisons of these ratings are reported in the Supplementary Materials (Table S3). Cognitive assessments were conducted at five time points after the intervention: immediately after stimulation (0 h) and after 1 h, 2 h, 4 h, and 8 h. At each time point, all participants completed the Attention Network Test (ANT, 10 min), the Cued Switching Task (CST, 5 min), and the 2-back task (5 min) in the same order, with the task order rotated across the five time points following a Latin-square design; task order was therefore identical for the two groups at every time point and is not confounded with group assignment. During the 8 h observation window, participants remained in the laboratory between assessments: mobile phones and other electronic devices were prohibited, sleeping and strenuous physical activity were not allowed, and reading, conversation, snacks, and water were permitted; lunch was standardized across participants (provided by the institutional canteen). The experimental procedure is illustrated in Figure 1.

### 2.3. Measures

**Figure 2:**
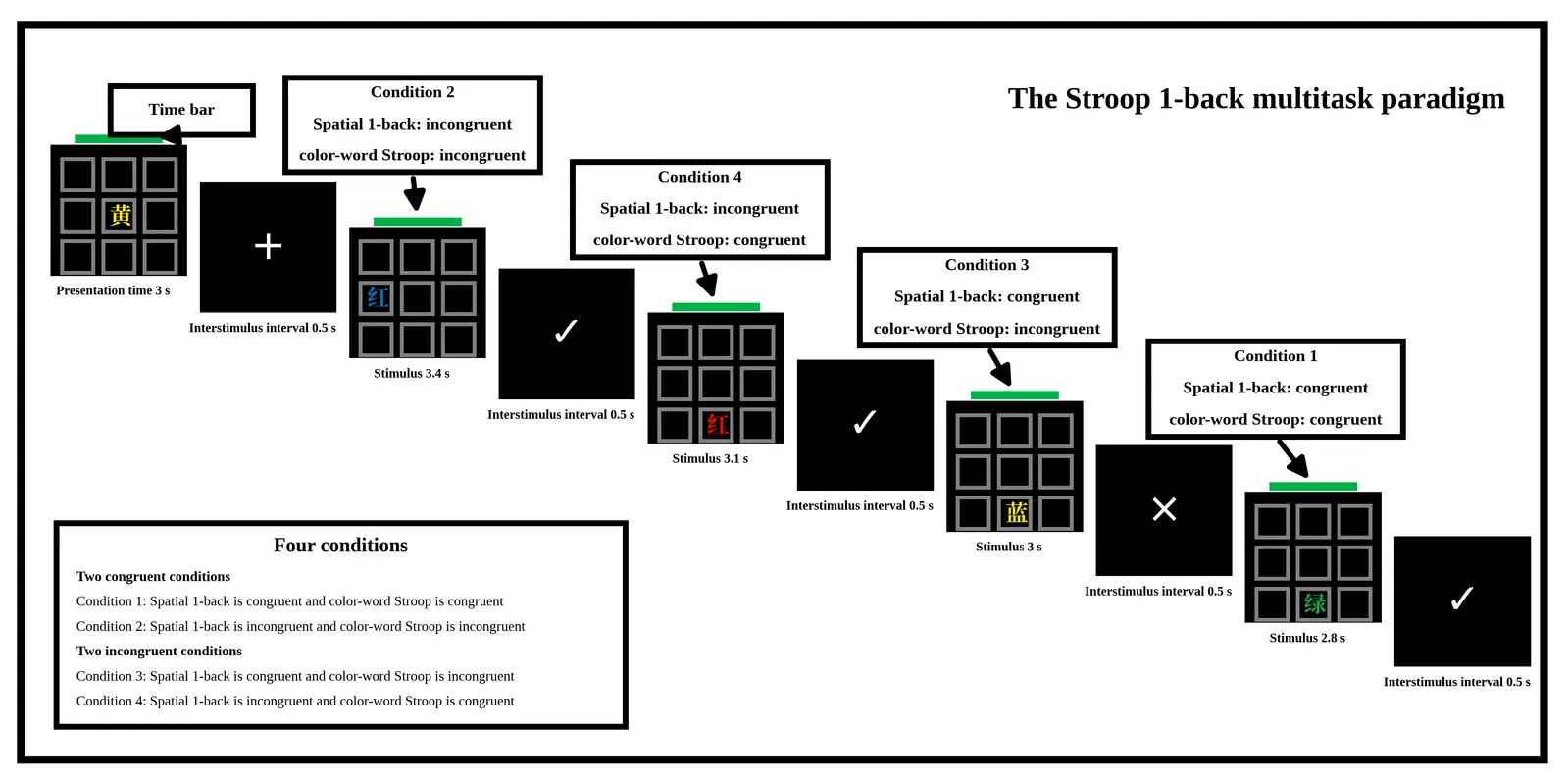
1-back Stroop task (BS). The panels are original screenshots of the experimental program. The colored Chinese characters denote color names (红 = red, 绿 = green, 蓝 = blue, 黄 = yellow), printed in either congruent or incongruent ink colors. The gray box marks the stimulus position on the previous trial (spatial 1-back reference); the green bar indicates the response deadline; “+” denotes the fixation point; “√” and “×” indicate correct and incorrect, respectively.

BS high-cognitive-load task: The BS task integrates two cognitive demands—spatial 1-back working memory and color–word Stroop interference—yielding four trial conditions (dual-congruent, position-congruent only, color-congruent only, and dual-incongruent) corresponding to two key responses [21]. The task interface consisted of a 3 × 3 grid of nine square spatial positions. In each trial, a colored Chinese character (printed in red, green, blue, or yellow; the character itself being “红” [red], “绿” [green], “蓝” [blue], or “黄” [yellow]) was randomly presented in one of the positions. Participants were required to make two simultaneous judgments: whether the spatial position of the current character matched that of the preceding trial (spatial 1-back), and whether the text color of the character matched its semantic meaning (color–word Stroop). Key responses were required based on these judgments: participants pressed the “F” key when the two judgments were consistent with each other (Conditions 1 and 2) and the “J” key when they were inconsistent (Conditions 3 and 4). The probabilities of congruent and incongruent trials were each 50% for both dimensions, and trial order was randomized. As a time-pressure manipulation, each trial had a condition-specific response deadline, displayed as a green progress bar (time bar) at the top of the screen: 2800 ms for Condition 1, 3400 ms for Condition 2, 3000 ms for Condition 3, and 3100 ms for Condition 4; the first stimulus of the task was presented for 3 s. Participants were instructed to respond as quickly and accurately as possible; after a keypress (or when the deadline elapsed), feedback was presented for 500 ms (“√” for correct, “×” for incorrect, and “–” for no response), and trials without a response before the deadline were scored as errors. The next trial appeared immediately after the feedback. The task comprised six 10 min blocks separated by 20 s breaks, for a total duration of 60 min. The task was programmed in E-Prime 3.0 (Psychology Software Tools, Sharpsburg, PA, USA) and presented on a 17-inch LCD monitor (Lenovo, Beijing, China; 60 Hz refresh rate) at a viewing distance of approximately 60 cm. One week before the formal experiment, participants completed a practice session of the task during the baseline phase to familiarize themselves with the task rules and minimize learning effects. Accuracy and reaction times were recorded, and block-wise changes in accuracy served as the behavioral index of high-cognitive-load induction; accuracy was computed as the number of correct keypress responses divided by the total number of trials. Accuracy, rather than reaction time, was used as the primary index because the time-pressure design of this paradigm truncates the reaction-time distribution, consistent with the original validation of the paradigm [21]. A schematic of the task is shown in Figure 2.

Attention Network Test (ANT): The classic paradigm developed by Fan et al. (2002) [23] was used to assess three attentional subcomponents: alerting, orienting, and executive control. On each trial, a target arrow was presented at the center of the screen, flanked by two arrows that were either congruent or incongruent with the target; participants judged the direction of the target arrow (left/right) and responded via keypress. The task included four cue conditions: no cue, center cue, double cue, and spatial cue, and two target conditions: congruent (flankers same direction as target) and incongruent (flankers opposite direction). Network efficiencies were computed as reaction time differences: alerting effect = no cue RT − double cue RT, orienting effect = center cue RT − spatial cue RT, and executive control effect (conflict effect) = incongruent RT − congruent RT. The task comprised 2 blocks with 144 trials total; participants could rest between blocks. Practice was completed one week before the formal experiment and was not included in the analysis.

Cued Switching Task (CST): The classic task-switching paradigm was used to measure cognitive flexibility and task-switching ability. Participants were required to make categorical judgments based on cue-induced rules, switching flexibly between two task rules: (1) Shape judgment: when the letter “S” appeared at the center of the screen, participants judged the shape of the subsequent figure (triangle: F key; circle: J key). (2) Color judgment: When the letter “C” appeared, participants judged the color of the subsequent figure (red: F key; green: J key). Each trial began with a cue letter (500 ms), followed by the target stimulus (1500 ms), and participants were instructed to respond as accurately and quickly as possible. Trial types were categorized as repeat trials (current cue identical to the previous trial) and switch trials (current cue different from the previous trial). Switch costs were calculated as switch trial reaction time minus repeat trial reaction time, baseline-corrected as the index of cognitive flexibility. The task comprised 1 practice block (10 trials) and 1 formal block of 100 trials presented continuously without sub-blocks, with switch and repeat trials each accounting for 50% of the formal trials in a program-randomized sequence; participants could rest after the block. Practice was completed one week before the formal experiment and was not included in the analysis.

A spatial 2-back task was used to assess working memory updating and maintenance [24]. The task interface consisted of a 3 × 3 grid with nine square positions at the center of the screen. On each trial, a black square appeared in one of the positions, and participants judged whether the current position was identical to that from two trials back (“same”: left key; “different”: right key). Stimulus duration was 500 ms, with an inter-stimulus interval (ISI) of 2000 ms. The task comprised 2 blocks with 100 trials total, with target (match) trials accounting for approximately one third of all trials (target:non-target ≈ 1:2), within the conventional range for n-back paradigms (25–33%). Accuracy and reaction time served as dependent variables.

NASA Task Load Index (NASA-TLX): Subjective workload was assessed using the classic multidimensional scale, which comprises six dimensions: (1) Mental Demand, (2) Physical Demand, (3) Temporal Demand, (4) Performance, (5) Effort, and (6) Frustration. Each dimension was rated on a 100 mm visual analogue scale divided into 20 gradations (5 mm intervals), yielding scores ranging from 0 to 20. An unweighted scoring procedure was adopted, and the mean of the six dimension scores (range: 0–20) was used as the index of overall subjective workload, with higher scores indicating greater perceived workload. The scale was administered twice on the experimental day—immediately before the BS task and immediately after its completion—to validate workload induction [25].

### 2.4. Statistical Analysis

All statistical analyses were performed using IBM SPSS Statistics 27.0 (IBM Corp., Armork, NY, USA), with the significance level set at α = 0.05 (two-tailed). Reaction-time analyses included correct trials only. Trials with reaction times below 200 ms (anticipatory responses) were excluded in all tasks; trials above 1500 ms were additionally excluded in the ANT, CST, and 2-back tasks. Given the study’s focus on executive control and cognitive flexibility, the conflict effect and the switch cost were designated as the two primary outcome domains, and their immediate (0 h) between-group effects were controlled for multiplicity using the Holm procedure; alerting, orienting, and 2-back outcomes were treated as secondary and exploratory. Model assumptions were examined via residual Q–Q plots, Shapiro–Wilk tests, residual-versus-fitted plots, and screening for influential observations (standardized residuals with |*z*| > 3); diagnostic results are reported in the Supplementary Materials (Figure S1). A comprehensive summary of model terms is provided in Supplementary Table S2.

#### 2.4.1. Statistical Analysis of Workload Induction

A 2 (Group: taVNS/Sham) × 6 (Block 1–6) repeated-measures ANOVA was conducted on task accuracy to examine changes in performance across the workload task and between-group differences; if Mauchly’s test indicated a violation of the sphericity assumption, Greenhouse–Geisser-corrected results were used. A 2 (Group) × 2 (Time: pre-/post-task) repeated-measures ANOVA was conducted on the mean NASA-TLX scores across the six dimensions (range: 0–20) to examine changes in subjective workload.

#### 2.4.2. Statistical Analysis of Intervention Effects

Linear mixed-effects models (LMMs) were preferred over repeated-measures ANOVA because they accommodate unequally spaced measurement occasions (0, 1, 2, 4, and 8 h), allow time and its quadratic term to be modeled as continuous variables, and retain participants with partially missing observations without listwise deletion. LMMs were fitted separately for the change scores of each cognitive outcome, defined as post-intervention measurements minus baseline values (change = post-intervention − baseline). Because the observation times were unequally spaced (0, 1, 2, 4, and 8 h) and the core research questions concerned the slope and curvature of the post-intervention trajectory, time was entered as a continuous variable, and a quadratic term (time^2^) was added to capture potential nonlinearity in the recovery process. A raw (uncentered) time parameterization was adopted, such that the group coefficient estimates the between-group difference immediately after the intervention (0 h), thereby testing the immediate effect of taVNS; the group × time and group × time^2^ interactions characterize the temporal features of the between-group difference (i.e., whether the between-group difference grows or attenuates over time). As sensitivity analyses, (i) an analogous LMM with time entered as a categorical factor was fitted to obtain per-time-point between-group contrasts of estimated marginal means (EMMs; Supplementary Table S1), and (ii) a time-weighted area-under-the-curve summary was computed for each participant using the trapezoidal rule over the unequally spaced occasions (0, 1, 2, 4, and 8 h) and divided by the 8 h span (mean time-weighted effect, MTE), which was compared between groups with independent-samples *t* tests. In addition, analyses of covariance (ANCOVAs) were conducted on the immediate (0 h) post-intervention raw scores of the two primary outcomes (conflict effect and switch cost), with the corresponding baseline value entered as a covariate. For each outcome, the fixed effects included intervention group (sham = 0, taVNS = 1), post-intervention time (in hours), the quadratic time term, and their interactions with group (group × time and group × time^2^). A subject-level random intercept was specified, with a variance–components (VC) covariance structure.

Parameters were estimated using restricted maximum likelihood (REML), and the significance of fixed effects was evaluated with Type III *F* tests. By estimating parameters from all available observations, mixed models make efficient use of data from participants with missing values under the missing-at-random (MAR) assumption, without requiring listwise deletion. Given that the attention networks, cognitive flexibility, and working memory represent theoretically distinct cognitive functions, each outcome was modeled independently. Denominator degrees of freedom were approximated using the Satterthwaite method, and the immediate (0 h) between-group difference was estimated from the group coefficient under the raw time parameterization. Effect sizes (Cohen’s *d*) for the immediate (0 h) effects were computed as the model-estimated 0 h between-group difference divided by the pooled within-group standard deviation of the 0 h change scores.

## 3. Results

### 3.1. Validation of High Cognitive Load Induction

The results of the behavioral validation are presented in Table 1. Mauchly’s test indicated that the sphericity assumption was violated for the Block main effect (*W* = 0.215, approximate χ^2^(14) = 89.330, *p* < 0.001); therefore, Greenhouse–Geisser-corrected results are reported. The corrected main effect of Block was significant, *F*(2.857, 171.416) = 10.491, *p* < 0.001, *η*^2^*p* = 0.149, with overall accuracy showing a general declining trend after reaching its peak at Block 2 (*M* = 0.915, *SE* = 0.010) and decreasing to its lowest level at Block 6 (*M* = 0.872, *SE* = 0.014). The main effect of Group was not significant, *F*(1, 60) = 0.001, *p* = 0.979, *η*^2^*p* < 0.001, nor was the Block × Group interaction (Greenhouse–Geisser corrected: *F*(2.857, 171.416) = 0.436, *p* = 0.718, *η*^2^*p* = 0.007). These results indicate that the two groups exhibited parallel performance decline during the induction phase, confirming successful induction of the high-cognitive-load state.

The results of the subjective validation are presented in Table 2. A repeated-measures ANOVA revealed a significant main effect of Time, *F*(1, 60) = 96.650, *p* < 0.001, *η*^2^*p* = 0.617: NASA-TLX scores in the taVNS group increased from *M* = 9.714 (*SE* = 0.476) before induction to *M* = 12.484 (*SE* = 0.416) after induction, and those in the sham group increased from *M* = 9.561 (*SE* = 0.456) to *M* = 11.922 (*SE* = 0.518). Neither the main effect of Group, *F*(1, 60) = 0.347, *p* = 0.558, *η*^2^*p* = 0.006, nor the Time × Group interaction, *F*(1, 60) = 0.616, *p* = 0.436, *η*^2^*p* = 0.010, was significant.

### 3.2. Intervention Effects

#### 3.2.1. Effects of taVNS on the Attention Networks

**Figure 3:**
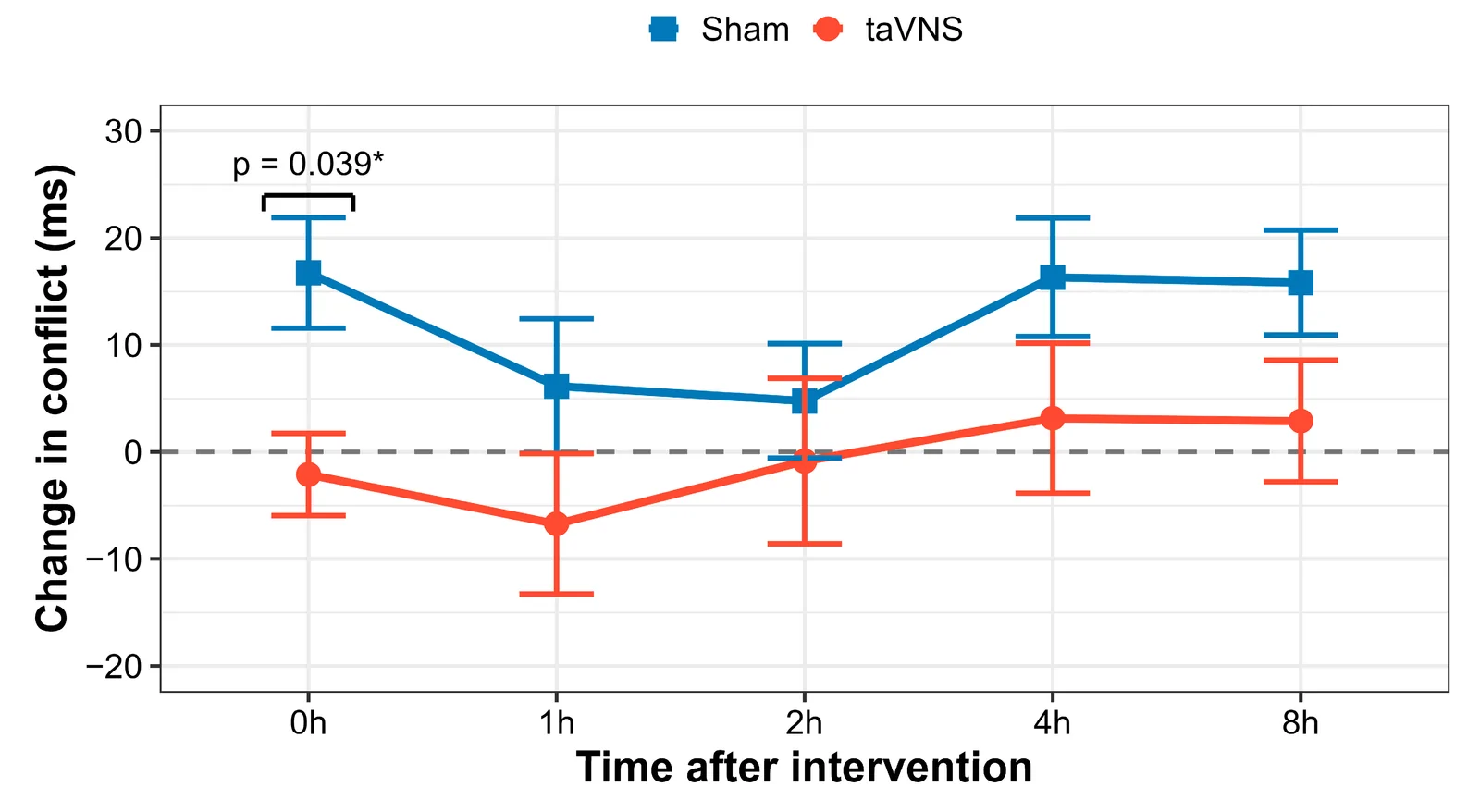
Changes in executive control network scores across post-intervention time points by group. Note. Change scores = post-intervention minus baseline; the dashed line denotes the baseline level (change = 0); error bars represent ±1 *SE*. * *p* < 0.05, significant between-group difference immediately after the intervention (0 h; based on the linear mixed-effects model: *b* = −16.43 ms, *p* = 0.039); the time-weighted AUC summary was directionally consistent but did not reach significance, *t*(60) = −1.75, *p* = 0.085.

For the alerting and orienting networks, the immediate (0 h) between-group differences were not significant (alerting: *b* = −1.04 ms, *SE* = 8.81, 95% CI [−18.49, 16.40], *F*(1, 122.0) = 0.01, *p* = 0.906; orienting: *b* = −2.50 ms, *SE* = 9.05, 95% CI [−20.40, 15.40], *F*(1, 133.8) = 0.08, *p* = 0.783); per-time-point between-group contrasts for all outcomes are reported in Supplementary Table S1. Neither the group × time nor the group × time^2^ interaction was significant for either network (*p*s > 0.05). In addition, for the alerting network, the main effect of time (*F*(1, 244) = 7.16, *p* = 0.008) and the main effect of time^2^ (*F*(1, 244) = 6.73, *p* = 0.010) were significant, with alerting-effect change scores in both groups showing an overall pattern of initial increase followed by a decline, although this temporal pattern did not differ between groups; for the orienting network, all time effects were nonsignificant, indicating flat trajectories. For the executive control network (see Table 3 and Figure 3), the LMM revealed a significant between-group difference in conflict-effect change scores immediately after the intervention (0 h), *b* = −16.43 ms, *SE* = 7.89, 95% CI [−32.03, −0.82], *F*(1, 127.5) = 4.34, *p* = 0.039, *d* = 0.64: the conflict effect increased from baseline in the sham group (*M* = 16.742 ± 5.183 ms) but decreased in the taVNS group (*M* = −1.087 ± 4.032 ms), indicating that taVNS improved executive control immediately after the intervention (Holm-adjusted *p* = 0.039). A baseline-adjusted ANCOVA on the 0 h post-intervention scores yielded a consistent result (adjusted between-group difference = −12.69 ms, 95% CI [−25.32, −0.06], *F*(1, 59) = 4.05, *p* = 0.049, partial *η*^2^ = 0.064). The time-weighted area-under-the-curve summary (mean time-weighted effect, MTE) was directionally consistent but did not reach statistical significance (taVNS 0.66 ± 30.28 ms vs. sham 12.79 ± 23.59 ms; between-group difference = −12.13 ms, 95% CI [−25.98, 1.72], *t*(60) = −1.75, *p* = 0.085, *d* = −0.44). The main effects of time and time^2^ and their interactions with group were not significant (*p*s > 0.05). Model-estimated between-group differences after 0, 1, 2, 4, and 8 h were −16.4, −14.0, −12.1, −10.0, and −12.0 ms, respectively; the categorical-time sensitivity analysis confirmed that the between-group difference was significant only after 0 h (Supplementary Table S1). Given the nonsignificant group × time interactions and the limited power of interaction tests, these estimates indicate an immediate effect of taVNS on executive control without establishing its persistence over the 8 h window.

#### 3.2.2. Effects of taVNS on the 2-Back Task

For accuracy change scores, the immediate (0 h) between-group difference was not significant (*b* = 0.011, *SE* = 0.015, 95% CI [−0.019, 0.041], *F*(1, 80.3) = 0.54, *p* = 0.463; per-time-point contrasts are reported in Supplementary Table S1); the main effects of time and time^2^ and the group × time and group × time^2^ interactions were also not significant (*p*s > 0.05).

For RT change scores, the immediate (0 h) between-group difference was not significant (*b* = 10.01 ms, *SE* = 26.94, 95% CI [−43.66, 63.68], *F*(1, 74.0) = 0.14, *p* = 0.711). The main effect of time was significant, *F*(1, 244) = 65.80, *p* < 0.001, as was the main effect of time^2^, *F*(1, 244) = 5.54, *p* = 0.019: RT change scores in both groups showed an overall pattern of rapid initial decline followed by a slight rebound (linear term *b* = −13.60; quadratic term *b* = 1.84), consistent with the typical shape of a saturating practice effect. Per-time-point contrasts are reported in Supplementary Table S1. Neither the group × time nor the group × time^2^ interaction was significant (*p*s > 0.05).

#### 3.2.3. Effects of taVNS on the CST

**Figure 4:**
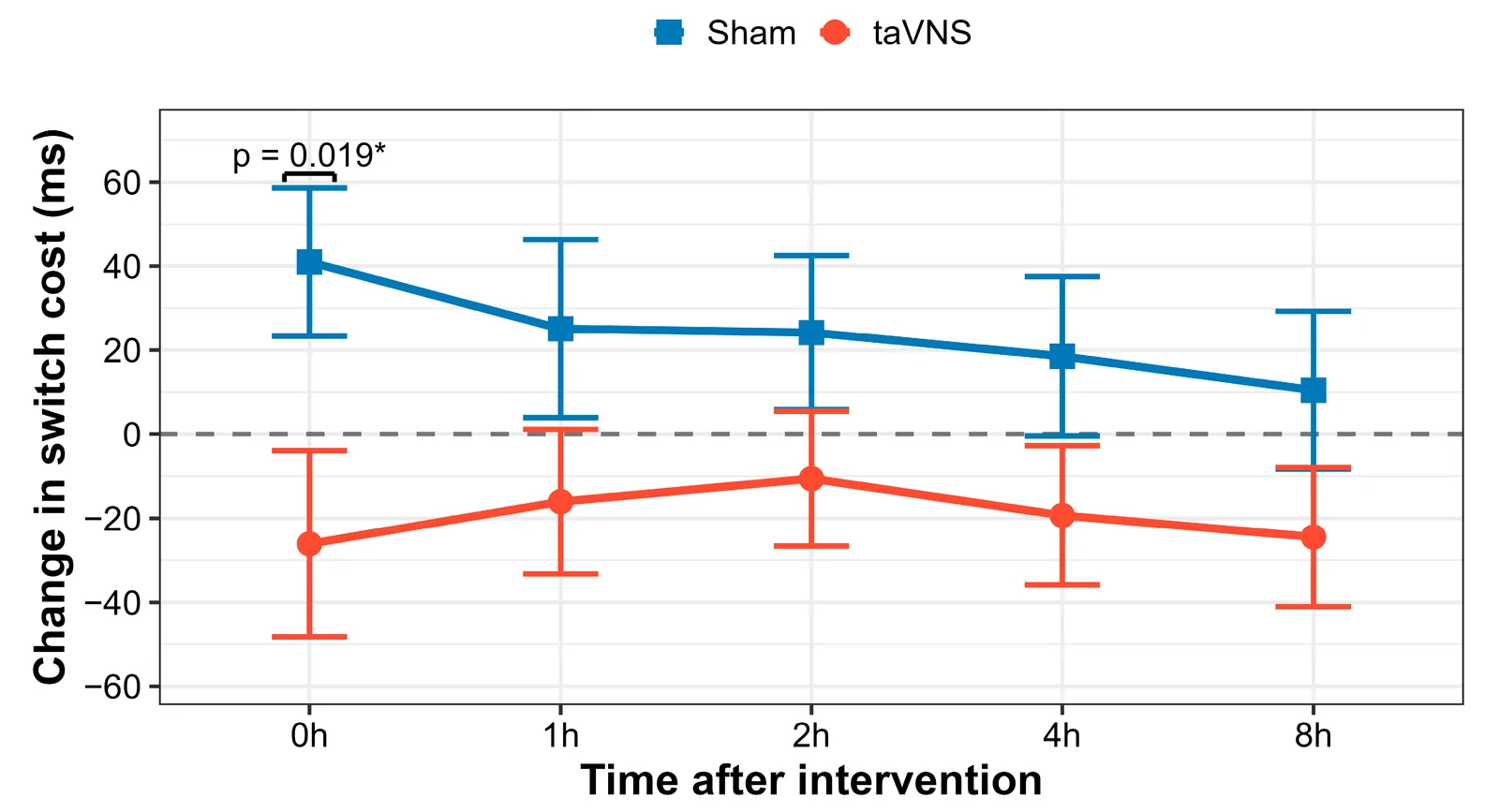
Changes in switch cost across post-intervention time points by group. Note. Change scores = post-intervention minus baseline; negative values indicate improved cognitive flexibility; the dashed line denotes the baseline level (change = 0); error bars represent ±1 *SE*. * *p* < 0.05, significant between-group difference immediately after the intervention (0 h; based on the LMM: *b* = −59.79 ms, *p* = 0.019); the time-weighted AUC summary was directionally consistent but did not reach significance, *t*(60) = −1.68, *p* = 0.098.

For switch-cost change scores (see Table 4 and Figure 4), the LMM revealed a significant between-group difference immediately after the intervention (0 h), *b* = −59.79 ms, *SE* = 25.07, 95% CI [−109.66, −9.92], *F*(1, 81.8) = 5.69, *p* = 0.019, *d* = 0.53: the switch cost increased from baseline in the sham group (*M* = 40.982 ± 17.641 ms) but decreased in the taVNS group (*M* = −26.106 ± 22.159 ms), indicating that taVNS improved cognitive flexibility immediately after the intervention (Holm-adjusted *p* = 0.038). Baseline-adjusted ANCOVA likewise confirmed the immediate effect (adjusted difference = −57.94 ms, 95% CI [−103.07, −12.81], *F*(1, 59) = 6.60, *p* = 0.013, partial *η*^2^ = 0.101). The time-weighted AUC summary (MTE) was directionally consistent but did not reach statistical significance (taVNS −18.98 ± 86.05 ms vs. sham 19.80 ± 95.64 ms; between-group difference = −38.78 ms, 95% CI [−84.94, 7.39], *t*(60) = −1.68, *p* = 0.098, *d* = −0.43). The main effects of time and time^2^ and their interactions with group were not significant (*p*s > 0.05). Model-estimated between-group differences after 0, 1, 2, 4, and 8 h were −59.8, −49.0, −40.5, −30.1, and −36.4 ms, respectively; the categorical-time sensitivity analysis confirmed significance only after 0 h (Supplementary Table S1), and the temporal course beyond the immediate effect should be interpreted cautiously.

#### 3.2.4. Tolerability and Blinding-Related Measures

No serious adverse events occurred. End-of-session sensation ratings (0–10) did not differ between groups for perceived stimulation intensity (taVNS 3.59 ± 1.66 vs. sham 2.77 ± 1.79, *p* = 0.065) or expected influence on subsequent performance (*p* = 0.292); headache ratings were minimal in both groups (0.16 ± 0.37 vs. 0.47 ± 0.86, *p* = 0.076). Blinding success was not formally assessed.

## 4. Discussion

The present study aimed to investigate the effects of transcutaneous auricular vagus nerve stimulation (taVNS) on attentional and executive functions following acute high cognitive load. A 1 h dual-task paradigm (1-back Stroop task) successfully induced a high-cognitive-load state, as evidenced by an overall declining trend after Block 2 and a significant increase in NASA-TLX subjective workload scores. On this basis, a randomized, single-blind, sham-controlled design was adopted to systematically evaluate the multidimensional effects of taVNS on the attention networks (alerting, orienting, and executive control), cognitive flexibility, and working memory. The core finding was that taVNS selectively improved the executive control network and cognitive flexibility, manifested as between-group separation immediately after the intervention, whereas no significant effects were observed for the alerting network, orienting network, or working memory. Three lines of evidence inform the temporal profile. First, the group × time interactions were nonsignificant; however, given the limited statistical power of interaction tests, this cannot be interpreted as evidence of equivalent recovery trajectories. Second, model-estimated between-group differences were directionally consistent across the 8 h observation window. Third, the categorical-time sensitivity analysis indicated significance only after 0 h, and the time-weighted AUC summaries did not reach significance. Together, these findings indicate that between-group separation was clearly present immediately after the intervention, whereas differences over longer time scales were not supported. Notably, the immediate effects on both primary outcomes remained significant in baseline-adjusted analyses of covariance (see Section 3), indicating that they were not driven by between-group differences in baseline performance. The core conclusion of the present study is therefore that taVNS produced a robust improvement at the end of the intervention; the implications of this immediate-onset pattern are elaborated below for each outcome.

taVNS significantly improved conflict monitoring following high cognitive load but had no significant effect on the alerting and orienting networks. This selectivity is consistent with the preferential prefrontal projections of the LC–NE system: vagal afferent fibers ascend via the nucleus tractus solitarius to the locus coeruleus, potentially activating noradrenergic neurons and thereby increasing norepinephrine release in the prefrontal cortex, particularly the anterior cingulate cortex (ACC) [11,26]—a mechanism proposed in the literature but not directly measured in the present study, which constitutes the core node of the executive control network [23]. In contrast, the alerting network relies on the thalamo-cortical system and the orienting network on the parietal cortex, both of which receive sparser LC–NE projections than the prefrontal cortex [27] and therefore respond weakly to taVNS. A recent meta-analysis by Liu and Li [28] found that taVNS produced a moderate effect on executive functions (Hedges’ g = 0.46) but only a small effect on working memory/attention (*g* = 0.19). The pattern observed in the present study—improved executive control but null alerting and orienting effects—aligns closely with these findings [28]. Notably, the improvement observed here was confined to the immediate post-intervention assessment: between-group contrasts beyond 0 h did not reach significance, and the time-weighted AUC summary was also nonsignificant. This temporal pattern is nonetheless informative in two respects. First, significant main effects of time were observed in both groups, indicating that the sham group gradually caught up through spontaneous recovery and that the between-group difference converged accordingly; this suggests that taVNS produced its clearest benefit in the immediate post-load period, when the performance cost of high cognitive load was greatest, consistent with prior evidence that non-invasive vagus nerve stimulation preserves cognitive performance under fatigued states such as sleep deprivation [29,30]. The benefit emerged precisely at the end of stimulation, when operational capability is most needed—a feature of particular practical value in sustained operations. Second, this pattern of immediate onset with subsequent convergence accords with the known action profile of vagus nerve stimulation: electrophysiological studies have shown that vagal stimulation rapidly drives locus coeruleus firing and elicits phasic norepinephrine release [31], and phasic LC–NE activation is thought to mediate rapid adjustments of cognitive state [27]. Given the limited power of the interaction tests, small persistent benefits beyond 0 h cannot be fully excluded; nevertheless, the immediate contrasts, the AUC summary, and the interaction tests jointly indicate a robust immediate benefit at the end of the 30 min stimulation period.

taVNS likewise improved cognitive flexibility: although the time-weighted (AUC) group difference in switch cost did not reach statistical significance (*p* = 0.098), the immediate post-intervention effect was robust (*p* = 0.019, *d* = 0.53), yielding a temporal pattern consistent with that of executive control—a significant immediate effect, with the between-group difference subsequently converging as the sham group recovered spontaneously. This cross-task consistency suggests that the effect of taVNS is not task-specific but a shared modulation of prefrontal executive networks. Mechanistically, cognitive flexibility relies on the task-switching network comprising the dorsolateral prefrontal cortex (dlPFC) and the ACC. The cue-switching task requires flexible alternation between task rules, a process that depends on rapid reconfiguration of cognitive control by the prefrontal cortex [32]. By enhancing norepinephrine release in the prefrontal cortex via the LC–NE system, taVNS may accelerate the flexible switching of task rule sets and reduce the “inertia” cost of cognitive control. Mena-Chamorro et al. found that the facilitating effect of taVNS on cognitive flexibility is more pronounced under high cognitive demand [20]. The present study observed a similar effect in the recovery context following high cognitive load, supporting a preferential modulation of taVNS for states following high cognitive load. Notably, Borges et al. in a randomized crossover trial, found that taVNS affected only specific subcomponents of cognitive flexibility (e.g., task-switching cost rather than set shifting), with small-to-medium effect sizes [18]. A meta-analysis also reported substantial between-study heterogeneity in taVNS effects on cognitive flexibility, suggesting that stimulation parameters and task characteristics may serve as important moderators [17]. Similar to the executive control network, the benefit for cognitive flexibility was likewise confined to the immediate post-intervention contrast. The two primary outcomes exhibited the same temporal pattern, suggesting that they may be regulated by a common mechanism, namely, the rapid adjustment of cognitive states by the LC–NE system [27,31]. It should be noted that the effect on switch cost rests primarily on the immediate post-intervention contrast: neither the time-weighted AUC summary (*p* = 0.098) nor the per-time-point contrasts beyond 0 h reached significance, and this effect should therefore be interpreted as an immediate improvement rather than a sustained one.

In contrast to the significant improvements in executive control and cognitive flexibility, taVNS produced no significant modulation of the alerting network, orienting network, or working memory; these null findings should not, by themselves, be taken as evidence of pathway specificity; several distinct factors may contribute. For the alerting network, the between-group difference in change scores was not statistically significant. The thalamo-cortical arousal system underlying the alerting network recovers relatively quickly [23], leaving limited room for additional taVNS gains—a “natural recovery ceiling.” Luna et al. further demonstrated that taVNS modulation of alerting is highly baseline-dependent, such that enhancement effects are difficult to observe when alertness is already high [33]. For the orienting network, the systematic review reported an effect size near zero [28]. Neuroanatomically, orienting primarily relies on the dorsal parietal–frontal attention network [34], and the parietal cortex receives sparser LC–NE projections than the prefrontal cortex [27]; taVNS-enhanced norepinephrine release therefore has limited influence on orienting. For working memory, neither the intervention protocol nor the task setting may have been optimal. Regarding the protocol, 25 Hz intermittent stimulation may be insufficient to activate working memory circuits, as Sun et al. found that 40 Hz burst stimulation significantly improved spatial working memory [35]. Dolphin et al. [11] noted considerable between-study variability in taVNS stimulation parameters (frequency, pulse width, and intensity), with cumulative stimulation dosage potentially affecting effect sizes. At the task level, the 2-back task imposes only moderate cognitive load, and evidence suggests that taVNS effects are more pronounced in more demanding paradigms such as 3-back or complex span tasks [36]. Collectively, the null results for these three indicators suggest that the cognitive enhancement effect of taVNS is not universal but is jointly moderated by the type of cognitive function, the natural recovery rate of the control condition, and the match between stimulation parameters and task characteristics.

In summary, taVNS holds potential as a cognitive countermeasure for high-demand operational settings, although this potential is currently supported only by laboratory evidence in healthy young men. First is immediate deployability: a 30 min intervention yields executive control improvements by its end, enabling rapid use during operational breaks, and is theoretically advantageous over caffeine (slow onset with side effects) and cognitive training (lengthy cycles) [37]. Second are portability and ease of use: the device is lightweight and self-administrable, and the intermittent stimulation protocol has a favorable safety profile. Third is functional targeting: taVNS improves the cognitive components that are least likely to recover spontaneously and most critical to task performance, namely inhibitory control and cognitive flexibility. Nevertheless, several limitations should be acknowledged. First, only behavioral measures were employed, without electrophysiological or neuroimaging assessments of taVNS effects on the LC–NE system and prefrontal activity, and the fixed 25 Hz intermittent protocol may not be optimal for functions such as working memory. Future studies should integrate neural measurements with parametric designs to identify optimal stimulation conditions for different cognitive functions. Second, the long-term effects and ecological validity of a single intervention remain limited: only the immediate and short-term effects of one 30 min taVNS session were assessed, without examining the cumulative effects of repeated interventions. Moreover, the 1 h laboratory workload differs from real combat scenarios, and the potential of taVNS under longer-duration cognitive loads warrants further investigation. Third, a formal assessment of blinding integrity was not implemented; however, end-of-session questionnaire ratings showed no significant between-group differences in perceived stimulation intensity or in the expected impact of stimulation on subsequent performance (see Supplementary Materials, Table S3), and all outcomes were objective, automatically recorded behavioral measures; nevertheless, as the researchers conducting testing and data analysis were aware of group allocation, expectancy-related influences cannot be fully excluded. Fourth, the stimulation device did not display or record absolute current intensity, precluding dosimetric reporting. Fifth, only healthy young men were recruited; generalizability to female populations requires direct investigation.

## 5. Conclusions

This study demonstrated that taVNS produced domain-specific cognitive benefits following sustained high cognitive load, selectively improving executive control and cognitive flexibility while showing no significant effects on alerting, orienting, or working memory. Although participants receiving taVNS exhibited better post-intervention performance than those receiving sham stimulation, the temporal pattern of post-intervention changes did not differ between groups. These findings provide preliminary evidence supporting the potential application of taVNS as a portable neuromodulation approach for cognitively demanding operational settings. Future studies should optimize stimulation parameters, investigate the underlying neural mechanisms, and evaluate the effectiveness of taVNS in more ecologically valid operational environments.

## Figures and Tables

**Table 1:** Task accuracy across blocks and repeated-measures ANOVA results by group (*M* ± *SE*).

	taVNS (*n* = 32)	Sham (*n* = 30)	Total (*N* = 62)
Block 1	0.910 ± 0.014	0.905 ± 0.015	0.908 ± 0.010
Block 2	0.919 ± 0.014	0.910 ± 0.014	0.915 ± 0.010
Block 3	0.910 ± 0.011	0.909 ± 0.019	0.910 ± 0.011
Block 4	0.899 ± 0.015	0.905 ± 0.016	0.902 ± 0.011
Block 5	0.876 ± 0.020	0.886 ± 0.017	0.881 ± 0.013
Block 6	0.871 ± 0.019	0.873 ± 0.022	0.872 ± 0.014
Source	*F* (df)	*p*	*η^2^p*
Block (G–G)	10.491 (2.857, 171.416)	<0.001	0.149
Group	0.001 (1, 60)	0.979	<0.001
Block × Group (G–G)	0.436 (2.857, 171.416)	0.718	0.007

Note. G–G = Greenhouse–Geisser-corrected.

**Table 2:** NASA-TLX scores before and after workload induction and repeated-measures ANOVA results by group (*M* ± *SE*).

	taVNS (*n* = 32)	Sham (*n* = 30)	Total (*N* = 62)
Pre	9.714 ± 0.476	9.561 ± 0.456	9.640 ± 0.327
Post	12.484 ± 0.416	11.922 ± 0.518	12.212 ± 0.329
Source	*F* (df)	*p*	*η^2^p*
Time	96.650 (1, 60)	<0.001	0.617
Group	0.347 (1, 60)	0.558	0.006
Time × Group	0.616 (1, 60)	0.436	0.010

Note. NASA-TLX scores are unweighted means of the six dimensions (range: 0–20), with higher scores indicating greater subjective workload.

**Table 3:** Descriptive statistics of change scores in the executive control network by group (*M* ± *SE*, ms).

	0 h	1 h	2 h	4 h	8 h
taVNS (*n* = 32)	−1.087 ± 4.032	−7.920 ± 7.587	−3.498 ± 6.994	3.498 ± 7.036	4.264 ± 6.051
Sham (*n* = 30)	16.742 ± 5.183	6.166 ± 6.301	4.775 ± 5.343	16.336 ± 5.557	15.821 ± 4.907

Note. Change scores = post-intervention values minus baseline; negative values indicate a reduced conflict effect relative to baseline (i.e., improved executive control). *SE* = standard error.

**Table 4:** Descriptive statistics of change scores in switch cost by group (*M* ± *SE*, ms).

	0 h	1 h	2 h	4 h	8 h
taVNS (*n* = 32)	−26.106 ± 22.159	−16.038 ± 17.205	−10.566 ± 16.068	−19.309 ± 16.530	−24.468 ± 16.551
Sham (*n* = 30)	40.982 ± 17.641	25.105 ± 21.163	24.190 ± 18.315	18.528 ± 18.978	10.476 ± 18.800

Note. Change scores = post-intervention minus baseline; negative values indicate a reduced switch cost relative to baseline (i.e., improved cognitive flexibility). *SE* = standard error.

## Data Availability

The data presented in this study are available on request from the corresponding author due to privacy and institutional restrictions.

## Supplementary Materials

The following supporting information can be downloaded at: https://www.mdpi.com/article/10.3390/brainsci16091001/s1, Figure S1: Residual diagnostic plots of the linear mixed models; Figure S2: Participant flow diagram (CONSORT format); Table S1: Between-group differences in change scores at each time point (taVNS—sham, based on a linear mixed model with categorical time points); Table S2: Fixed-effect estimates of the linear mixed models for each outcome (change scores); Table S3: Between-group comparison of end-of-session stimulation sensation questionnaire items.
