# Supplementary material for: Transcutaneous Auricular Vagus Nerve Stimulation Selectively Improves Executive Control and Cognitive Flexibility Following Sustained High Cognitive Load: A Randomized, Single-Blind, Sham-Controlled Study
Source: Brain Sci. 2026 Sep 21;16(9):1001. doi: 10.3390/brainsci16091001 (PMC13604627; doi:10.3390/brainsci16091001)
Supplement: Supplementary file 1 [file brainsci-16-01001-s001.zip › brainsci-4546203-supplementary.pdf]

Supplementary Materials: Between-Group Comparisons at Each Time Point and Model Diagnostics

Transcutaneous auricular vagus nerve stimulation selectively improves executive control and cognitive flexibility following sustained high cognitive load: a randomized, single-blind, sham-controlled study

1. Supplementary Analysis Methods

1.1. Between-Group Comparisons at Each Time Point (Sensitivity Analysis)

To examine between-group differences at each follow-up time point, a linear mixed model with time point as a categorical variable (0, 1, 2, 4, and 8 h) was fitted in addition to the primary analysis (a linear mixed model with time as a continuous variable): fixed effects included group, time point (categorical), and their interaction, with a random intercept for participants; denominator degrees of freedom were estimated using the Satterthwaite approximation. Based on this model, estimated marginal means (EMMs) of the change scores (relative to baseline) for both groups at each time point were calculated, and simple between-group comparisons were performed at each time point. Because this analysis served as a sensitivity check of the primary results rather than an independent hypothesis test, no further multiplicity correction was applied to the per-time-point comparisons (LSD). All analyses were performed in SPSS (MIXED procedure), with two-tailed $\alpha = .05$.

1.2. Model Diagnostics

Residual diagnostics were performed for the linear mixed model of each outcome: residual normality was examined with Q–Q plots and the Shapiro–Wilk test, homogeneity of variance was examined with residual-versus-fitted scatter plots, and outlying observations with standardized residuals $|z| > 3$ were screened. For the conflict effect model, the Shapiro–Wilk test gave $W = .996$, $p = .636$; the Q–Q plot approximated the diagonal line; the residual-versus-fitted scatter plot showed no obvious funnel shape; and no marked violations of the normality or homoscedasticity assumptions were found. Only 1 of 310 observations (0.3%) had a standardized residual $|z| > 3$, and including or excluding this observation did not change the statistical conclusions. Diagnostics for the switch cost model were consistent: Shapiro–Wilk $W = .992$, $p = .117$; the residual-versus-fitted scatter plot showed no obvious funnel shape; 3 of 310 observations (1.0%) had standardized residuals $|z| > 3$; and after these were excluded and the model was refitted, the between-group difference at 0 h remained significant ($p = .035$), leaving the conclusions unchanged. Neither model had missing data (310/310). The model diagnostic plots are shown in Figure S1.

2. Supplementary Results

Between-group differences (taVNS − sham) and 95% confidence intervals for all six outcomes at each time point are shown in Table S1.

Executive control (conflict effect). Immediately after the intervention (0 h), the change score of the conflict effect in the taVNS group was significantly lower than that in the sham group (difference = −17.83 ms, 95% CI [−34.67, −0.99], $F(1, 161.9) = 4.37$, $p = .038$). At this time point, the EMM of the sham group was 16.74 ms (95% CI [4.65, 28.84]; the confidence interval did not include 0, i.e., the conflict effect remained significantly above baseline after sham stimulation), whereas the EMM of the taVNS group was −1.09 ms (95% CI [−12.80, 10.63]; the confidence interval included 0, i.e., no significant difference from baseline). Between-group differences at all subsequent time points were not significant (1 h: $p = .100$; 2 h: $p = .333$; 4 h: $p = .134$; 8 h: $p = .177$). This time-point pattern is consistent with the primary analysis: a clear between-group separation existed immediately after the intervention, and between-group differences were not significant at any subsequent time point.

Cognitive flexibility (switch cost). Immediately after the intervention (0 h), the change score of the switch cost in the taVNS group was significantly lower than that in the sham group (difference = −67.09 ms, 95% CI [−118.81, −15.37], $F(1, 94.5) = 6.63$, $p = .012$; sham group EMM = 40.98 ms, 95% CI [3.82, 78.14]); between-group differences at the remaining time points were not significant (1 h: $p = .118$; 2 h: $p = .185$; 4 h: $p = .150$; 8 h: $p = .183$).

Alerting and orienting. The two groups showed no significant between-group differences in the change scores of the alerting effect ($p \geq .269$ at all time points) or the orienting effect ($p \geq .220$ at all time points), consistent with the primary analysis, in which no significant between-group differences were found.

Working memory (2-back). The two groups showed no significant between-group differences in the change scores of 2-back accuracy ($p \geq .327$ at all time points) or reaction time ($p \geq .103$ at all time points), consistent with the primary analysis.

3. Supplementary Tables

Table S1. Between-group differences in change scores at each time point (taVNS − sham, based on a linear mixed model with categorical time points)

Outcome	Time point (h)	Difference	*SE*	df	*F*	*p*	95% CI Lower	95% CI Upper
Conflict effect (ms)	0	**−17.83**	8.53	161.9	4.37	**.038**	−34.67	−0.99
Conflict effect (ms)	1	−14.09	8.53	161.9	2.73	.100	−30.92	2.75
Conflict effect (ms)	2	−8.27	8.53	161.9	0.94	.333	−25.11	8.57
Conflict effect (ms)	4	−12.84	8.53	161.9	2.27	.134	−29.68	4.00
Conflict effect (ms)	8	−11.56	8.53	161.9	1.84	.177	−28.40	5.28
Alerting (ms)	0	−5.73	9.51	155.1	0.36	.547	−24.52	13.05
Alerting (ms)	1	10.56	9.51	155.1	1.23	.269	−8.23	29.35
Alerting (ms)	2	−7.70	9.51	155.1	0.65	.420	−26.48	11.09
Alerting (ms)	4	−0.41	9.51	155.1	0.00	.966	−19.20	18.38

Outcome	Time point (h)	Difference	*SE*	df	*F*	*p*	95% CI Lower	95% CI Upper
Alerting (ms)	8	−3.88	9.51	155.1	0.17	.684	−22.67	14.91
Orienting (ms)	0	−2.09	9.87	172.6	0.04	.833	−21.57	17.39
Orienting (ms)	1	−2.24	9.87	172.6	0.05	.821	−21.71	17.24
Orienting (ms)	2	12.15	9.87	172.6	1.52	.220	−7.33	31.63
Orienting (ms)	4	5.87	9.87	172.6	0.35	.553	−13.61	25.34
Orienting (ms)	8	1.58	9.87	172.6	0.03	.873	−17.90	21.05
Switch cost (ms)	0	**−67.09**	26.05	94.5	6.63	**.012**	−118.81	−15.37
Switch cost (ms)	1	−41.14	26.05	94.5	2.49	.118	−92.87	10.58
Switch cost (ms)	2	−34.76	26.05	94.5	1.78	.185	−86.48	16.97
Switch cost (ms)	4	−37.84	26.05	94.5	2.11	.150	−89.56	13.89
Switch cost (ms)	8	−34.94	26.05	94.5	1.80	.183	−86.67	16.78
2-back accuracy	0	0.02	0.02	134.3	0.89	.327	−0.02	0.05
2-back accuracy	1	0.00	0.02	134.3	0.00	.965	−0.03	0.04
2-back accuracy	2	0.02	0.02	134.3	0.89	.346	−0.02	0.05
2-back accuracy	4	0.01	0.02	134.3	0.60	.415	−0.02	0.05
2-back accuracy	8	0.02	0.02	134.3	0.79	.379	−0.02	0.05
2-back RT (ms)	0	−0.66	29.79	107.6	0.00	.982	−59.72	58.39
2-back RT (ms)	1	15.73	29.79	107.6	0.28	.599	−43.32	74.79
2-back RT (ms)	2	−14.02	29.79	107.6	0.22	.639	−73.07	45.04
2-back RT (ms)	4	−6.86	29.79	107.6	0.05	.818	−65.91	52.20
2-back RT (ms)	8	−49.01	29.79	107.6	2.71	.103	−108.06	10.05

Note: Based on a linear mixed model with group × time point (categorical) as fixed effects and a random intercept for participants; denominator degrees of freedom were estimated using the Satterthwaite approximation; differences are the differences in estimated marginal means between the two groups (taVNS − sham), and the dependent variable is the change score relative to baseline. Bold indicates $p < .05$.

Table S2. Fixed-effect estimates of the linear mixed models for each outcome (change scores)

**Outcome**	**Model term**	***b***	***SE***	**df**	***F***	***p***	**95% CI Lower**	**95% CI Upper**
Conflict effect (ms)	Intercept	11.78	5.67	127.5	0.82	.367	0.57	22.99
	Group	−16.43	7.89	127.5	4.34	.039	−32.03	−0.82
	Time	−1.35	2.73	244	0.00	.997	−6.72	4.03
	Time²	0.25	0.32	244	0.27	.605	−0.38	0.88
	Group × Time	2.68	3.80	244	0.50	.481	−4.80	10.16
	Group × Time²	−0.27	0.44	244	0.36	.550	−1.14	0.61
	Intercept	10.06	6.33	122.0	4.68	.032	−2.48	22.59

Alerting effect (ms)	Group	−1.04	8.81	122.0	0.01	.906	−18.49	16.40
	Time	5.29	2.97	244	7.16	.008	−0.55	11.14
	Time²	−0.57	0.35	244	6.73	.010	−1.26	0.11
	Group × Time	0.46	4.13	244	0.01	.910	−7.67	8.60
	Group × Time²	−0.10	0.48	244	0.05	.828	−1.06	0.85
Orienting effect (ms)	Intercept	3.07	6.50	133.8	0.16	.688	−9.79	15.93
	Group	−2.50	9.05	133.8	0.08	.783	−20.40	15.40
	Time	−4.57	3.22	244	0.76	.384	−10.92	1.77
	Time²	0.49	0.38	244	0.53	.469	−0.25	1.23
	Group × Time	5.23	4.48	244	1.36	.245	−3.60	14.06
	Group × Time²	−0.60	0.52	244	1.29	.256	−1.63	0.44
Switch cost (ms)	Intercept	37.34	18.01	81.8	0.35	.554	1.52	73.17
	Group	−59.79	25.07	81.8	5.69	.019	−109.66	−9.92
	Time	−7.42	5.75	244	0.14	.714	−18.74	3.90
	Time²	0.52	0.67	244	0.01	.924	−0.81	1.84
	Group × Time	11.91	8.00	244	2.22	.138	−3.85	27.67
	Group × Time²	−1.12	0.94	244	1.44	.231	−2.97	0.72
2-back accuracy	Intercept	−0.0263	0.0109	80.3	7.44	.008	−0.0480	−0.0046
	Group	0.0112	0.0152	80.3	0.54	.463	−0.0190	0.0414
	Time	0.0013	0.0019	244	1.29	.257	−0.0024	0.0050
	Time²	0.0006	0.0006	244	2.48	.116	−0.0005	0.0018
	Group × Time	0.0004	0.0026	244	0.03	.873	−0.0047	0.0056
	Group × Time²	0.0001	0.0008	244	0.01	.944	−0.0016	0.0017
2-back RT (ms)	Intercept	−64.29	19.35	74.0	19.38	<.001	−102.85	−25.73
	Group	10.01	26.94	74.0	0.14	.711	−43.66	63.68

Time	−13.60	2.85	244	65.80	<.001	−19.22	−7.98
Time^2	1.84	0.90	244	5.54	.019	0.07	3.61
Group × Time	−5.02	3.97	244	1.60	.208	−12.84	2.80
Group × Time^2	−0.74	1.25	244	0.35	.554	−3.20	1.72

Note: The dependent variable is the change score of each outcome (post-intervention − baseline); fixed effects include group (with sham as the reference; under the original time parameterization, the group coefficient is the between-group difference at 0 h immediately after the intervention), time (hours), the quadratic time term, and their interactions with group; the random effect is a random intercept for participants (variance components structure); denominator degrees of freedom were estimated using the Satterthwaite approximation; b, SE, and 95% CIs are from the fixed-effect estimates, and F and p values are from type III tests of fixed effects; millisecond outcomes are reported to two decimal places, and 2-back accuracy to four decimal places.

Table S3. Between-group comparison of end-of-session stimulation sensation questionnaire items

Item	taVNS group (*n* = 32)	Sham group (*n* = 30)	***t***	**df**	***p***
Visual perceptual changes (e.g., blurred vision, flashes)	1.78 ± 1.68	2.87 ± 2.53	−1.977	49.96	.054
Acute mood changes (e.g., sudden elevation or lowering of mood)	2.56 ± 2.26	2.03 ± 2.04	0.969	59.93	.336
Fatigue relief	3.09 ± 1.35	2.77 ± 1.48	0.907	58.61	.368
Headache	0.16 ± 0.37	0.47 ± 0.86	−1.825	38.78	.076
Difficulty concentrating	2.94 ± 1.76	2.30 ± 1.54	1.523	59.71	.133
Perceived stimulation intensity (faint to strong)	3.59 ± 1.66	2.77 ± 1.79	1.879	58.83	.065
Expected influence of stimulation on subsequent performance	2.97 ± 1.88	2.50 ± 1.59	1.063	59.44	.292

Note: The questionnaire was completed at the end of the experimental session (after the final 8-h assessment); each item was rated on a 0–10 scale; values are means ± SDs; between-group comparisons used independent-samples *t* tests with the Welch correction (not assuming equal variances).

4. Supplementary Figures

Figure S1. Residual diagnostic plots of the linear mixed models

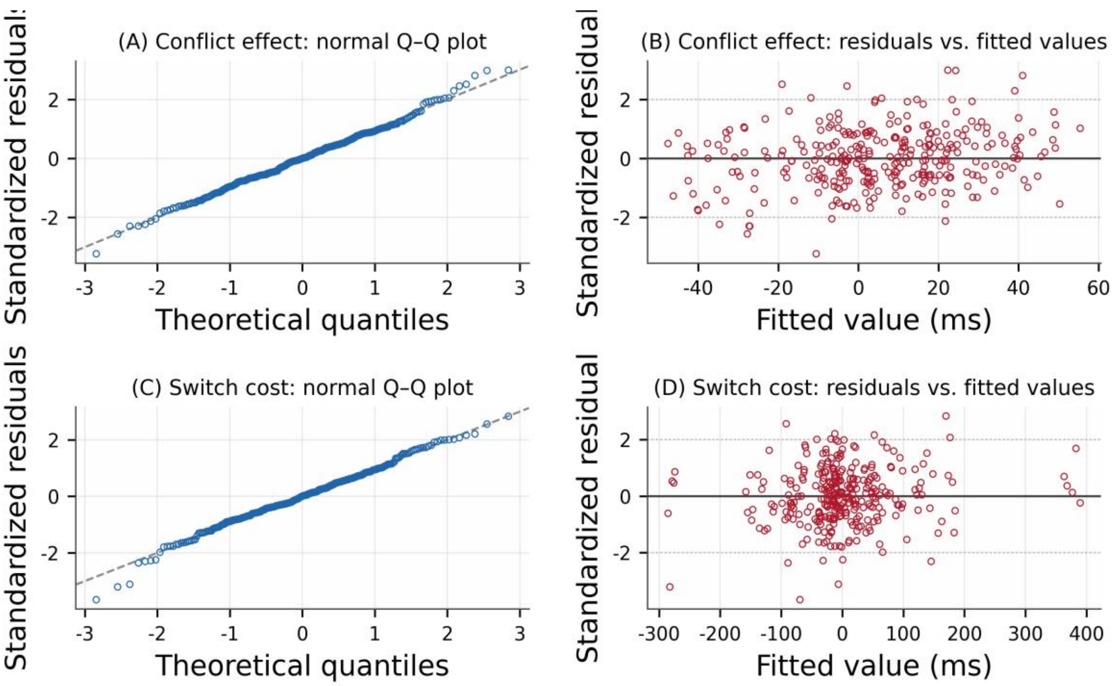

Figure S1

Caption: (A) and (C) are normal Q–Q plots of standardized residuals, with points approximately distributed along the diagonal, supporting the residual normality assumption; (B) and (D) are residual-versus-fitted scatter plots, with no funnel-shaped distribution, supporting the homoscedasticity assumption. Conflict effect model: Shapiro–Wilk $W = .996$, $p = .636$; switch cost model: $W = .992$, $p = .117$.

Figure S2. *Participant flow diagram (CONSORT format)*

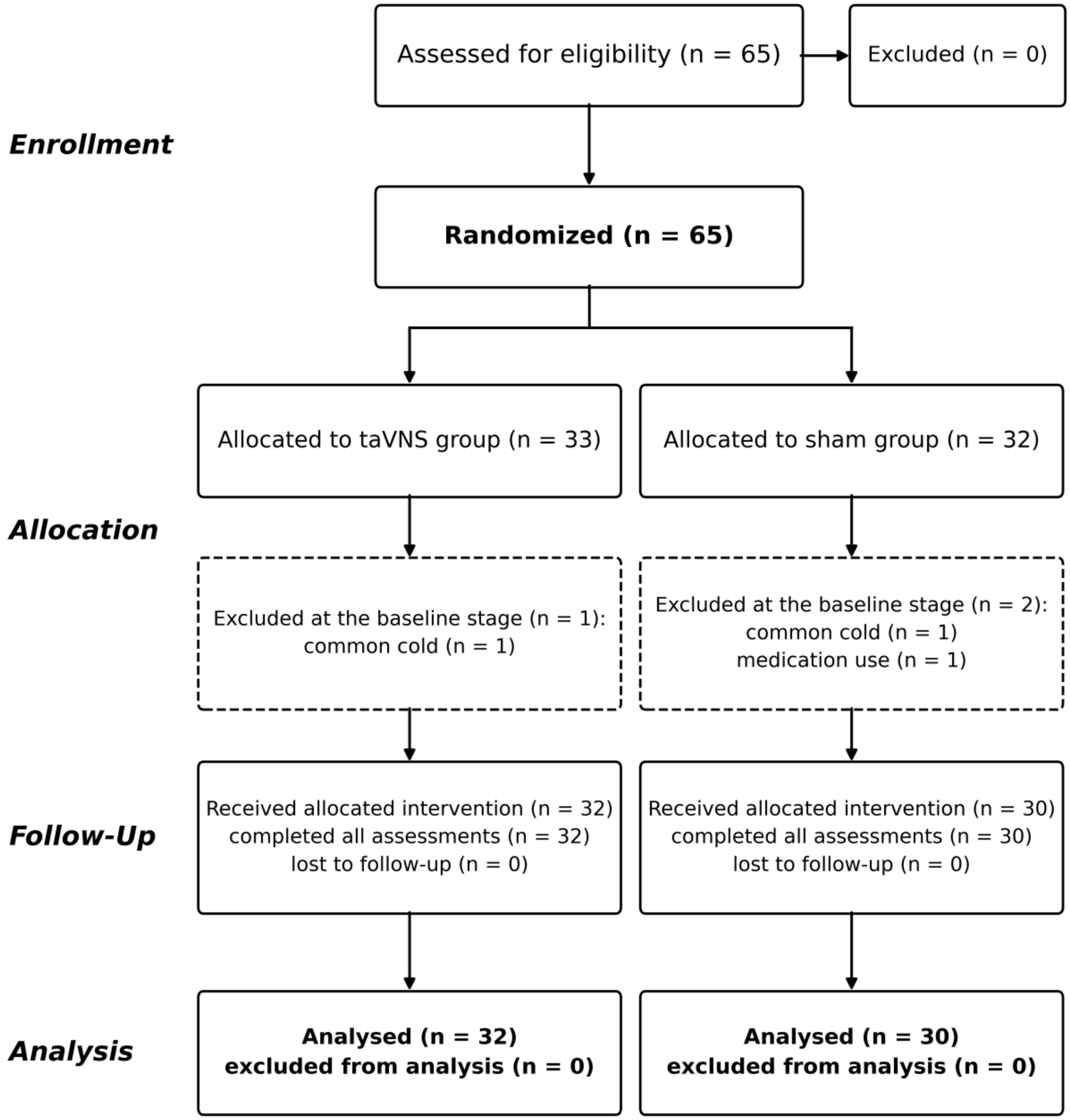

Caption: All 65 assessed participants met the inclusion criteria and were randomized (33 to the taVNS group and 32 to the sham group); 3 participants were excluded at the baseline stage (1 in the taVNS group due to a common cold during baseline testing, and 2 in the sham group—1 with a common cold and 1 due to concomitant medication use); the final 62 participants (32 in the taVNS group and 30 in the sham group) completed all five post-intervention assessments and were included in the analysis, with no loss to follow-up.
